# Global research trends on COVID-19 and stroke: A bibliometric analysis

**DOI:** 10.3389/fneur.2023.1147867

**Published:** 2023-04-03

**Authors:** Youjie Zeng, Si Cao, Heng Yang

**Affiliations:** ^1^Department of Anesthesiology, Third Xiangya Hospital, Central South University, Changsha, Hunan, China; ^2^Department of Neurology, Third Xiangya Hospital, Central South University, Changsha, Hunan, China

**Keywords:** COVID-19, stroke, bibliometric analysis, Web of Science, VOSviewer, Citespace, research hotspots, visual analysis

## Abstract

**Background:**

The pandemic of COVID-19 has had a profound influence on worldwide healthcare systems. Our study aimed to conduct a bibliometric analysis to explore the impact of COVID-19 on stroke and to highlight the major research trends in this field.

**Methods:**

We searched the original articles and review articles regarding COVID-19 and stroke from the Web of Science collection (WOSCC) database between January 1, 2020 and December 30, 2022. Subsequently, we performed bibliometric analyses and visualization using VOSviewer, Citespace, and Scimago Graphica.

**Results:**

A total of 608 original articles or review articles were included. JOURNAL OF STROKE and CEREBROVASCULAR DISEASES published the most studies on this subject (*n* = 76), while STROKE was the source of the most-cited references (*n* = 2,393). The United States is the most influential country in this field, with the highest number of publications (*n* = 223) and citations (*n* = 5,042). Shadi Yaghi from New York University is the most prolific author in the field, while Harvard Medical School is the most prolific institution. In addition, through keyword analysis and reference co-citation analysis, three major research topics were identified: (i) the impact of COVID-19 on stroke outcomes (including risk factors, clinical characteristics, mortality, stress, depression, comorbidities, etc.); (ii) the management and care of stroke patients during the COVID-19 pandemic (including thrombolysis, thrombectomy, telemedicine, anticoagulation, vaccination, etc.); and (iii) the potential relationship and pathological mechanism between COVID-19 and stroke (including renin-angiotensin system activation, SARS-CoV-2 virus-induced inflammation leading to endothelial impairment, coagulopathy, etc.).

**Conclusion:**

Our bibliometric analysis provides a comprehensive overview of the current state of research on COVID-19 and stroke and highlights key areas of focus in the field. Optimizing the treatment of COVID-19-infected stroke patients and elucidating the underlying pathogenic mechanisms of COVID-19 and stroke co-morbidity are key areas of future research that will be beneficial in improving the prognosis of stroke patients during the ongoing COVID-19 epidemic.

## 1. Introduction

The COVID-19 pandemic, triggered by the new coronavirus SARS-CoV-2, has posed unprecedented threats to global health and economies (1). Over the past 3 years, the World Health Organization has documented 759 million confirmed cases of COVID-19 and over 6 million fatalities (https://covid19.who.int, accessed on March 14, 2022). The most common clinical manifestations of COVID-19 infection are respiratory symptoms, such as dry cough, fever, and breathing difficulties (2). However, in addition to respiratory symptoms, COVID-19 has been linked to various other health complications, including neurological and cardiovascular issues (3, 4).

Studies have suggested an association between COVID-19 and stroke. Individuals with COVID-19 have an increased risk of stroke, especially those with severe COVID-19 infections (5, 6). It has been discovered that COVID-19 patients had higher levels of coagulation factors and systemic inflammation (7, 8), both of which are known risk factors for stroke (9, 10). In addition, COVID-19 may worsen various risk factors for stroke, such as hypertension and diabetes (11). Furthermore, COVID-19 patients with stroke were more likely to experience poor outcomes and prolonged hospitalization (12).

Bibliometric analysis is a quantitative methodology that uses statistical tools to examine research works of literature on a particular topic (13). Previous bibliometric analyses have depicted research trends in neurologic and neurosurgical articles related to COVID-19, showcasing the effects of the COVID-19 pandemic on the field of neuroscience (14, 15). Nevertheless, the publications regarding COVID-19 and stroke have not been further investigated. Therefore, an in-depth analysis of the research trends involving COVID-19 and stroke is necessary. In the current study, we will use bibliometric techniques to explore the current state of research on COVID-19 and stroke. Specifically, we will evaluate the influential journals in COVID-19 and stroke research and determine the leading countries, authors, institutions, and publications in the field. In addition, we will conduct keyword and reference co-citation analysis to determine the main hot topics.

## 2. Materials and methods

**Figure 1:**
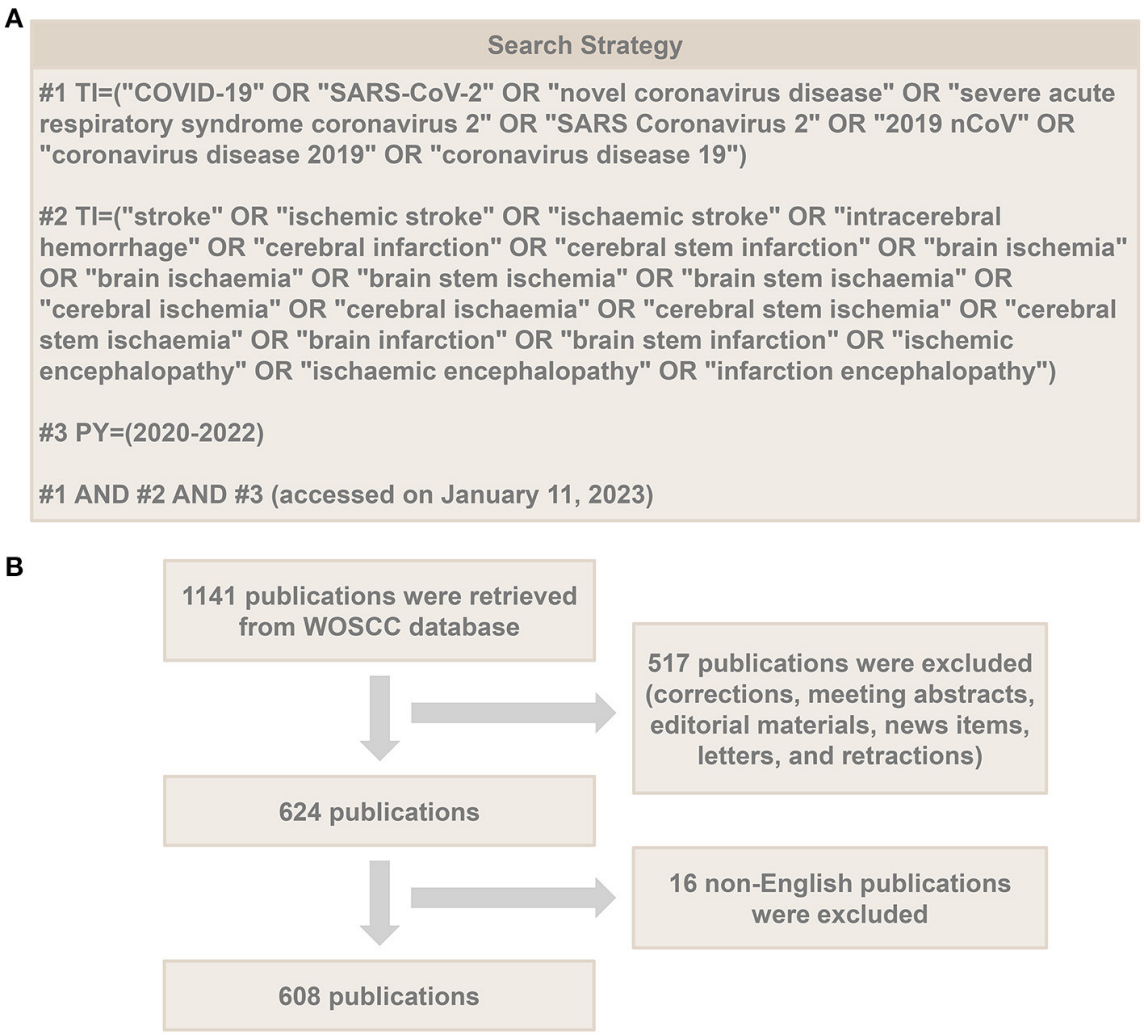
The data collection process of this study. **(A)** Searching strategy; **(B)** screening process.

The data were obtained from the SCI-Expanded edition of the Web of Science Core Collection (WoSCC) database on January 11, 2023. Search strategy: TI = (“COVID-19” OR “SARS-CoV-2” OR “novel coronavirus disease” OR “severe acute respiratory syndrome coronavirus 2” OR “SARS Coronavirus 2” OR “2019 nCoV” OR “coronavirus disease 2019” OR “coronavirus disease 19”) AND TI = (“stroke” OR “ischemic stroke” OR “ischaemic stroke” OR “intracerebral hemorrhage” OR “cerebral infarction” OR “cerebral stem infarction” OR “brain ischemia” OR “brain ischaemia” OR “brain stem ischemia” OR “brain stem ischaemia” OR “cerebral ischemia” OR “cerebral ischaemia” OR “cerebral stem ischemia” OR “cerebral stem ischaemia” OR “brain infarction” OR “brain stem infarction” OR “ischemic encephalopathy” OR “ischaemic encephalopathy” OR “infarction encephalopathy”) AND PY = (2020–2022). Subsequently, we only included articles and review articles, and excluded corrections, meeting abstracts, editorial materials, news items, letters, retractions and non-English publications. A total of 608 publications were eventually identified, including 518 original articles and 90 review articles. We then downloaded these records in plain text file format for subsequent analysis. In addition, the journal impact factor was retrieved from Thomson Reuters Journal citation reports 2021 *via* the Web of Science database. Figure 1 presents the data collection process of this study.

For data visualization in this work, VOSviewer (1.6.18), Scimago Graphica, and Citespace (6.1.R6) were utilized (16, 17). We employed VOSviewer for analyzing and visualizing influential journals, country/region co-authorship, author co-authorship, institution co-authorship, influential publications, and keyword co-occurrence. Meanwhile, Scimago Graphica was utilized to plot chord diagrams to depict collaboration between countries/regions, authors, and institutions. In addition, Citespace was used to conduct reference co-citation analysis to recognize major research areas.

## 3. Results

### 3.1. Overview of the included publications

We retrieved 608 qualified publications related to COVID-19 and stroke on January 11, 2023, including 518 original articles and 90 review articles. The 608 publications involved in this study were generated by 5,323 authors from 1,911 institutions located in 81 countries/regions. In addition, they were published in 189 journals and cited 10,342 references in 3,014 journals.

### 3.2. Top contributing and co-cited journals

Our bibliometric analysis included 608 publications published in 189 journals. Among these journals, ten released more than ten publications related to COVID-19 and stroke, namely JOURNAL OF STROKE and CEREBROVASCULAR DISEASES, STROKE, FRONTIERS IN NEUROLOGY, NEUROLOGICAL SCIENCES, EUROPEAN JOURNAL OF NEUROLOGY, CEREBROVASCULAR DISEASES, AMERICAN JOURNAL OF NEURORADIOLOGY, INTERNATIONAL JOURNAL OF STROKE, JOURNAL OF NEUROINTERVENTIONAL SURGERY, and JOURNAL OF NEUROLOGY (Table 1). The STROKE journal has the highest impact factor (IF) of the ten most productive journals (IF = 10.17).

**Table 1:** Top 10 productive and most-cited journals in COVID-19 and stroke research.

**Rank**	**Journal**	**NP (%)**	**IF (2021)**	**JCR**	**Cited journal**	**Citation frequency**	**IF (2021)**	**JCR**
1	JOURNAL OF STROKE and CEREBROVASCULAR DISEASES	76 (12.50 %)	2.677	Q3	STROKE	2,393	10.17	Q1
2	STROKE	59 (9.70 %)	10.17	Q1	NEW ENGLAND JOURNAL OF MEDICINE	984	176.082	Q1
3	FRONTIERS IN NEUROLOGY	42 (6.91 %)	4.086	Q2	JOURNAL OF STROKE and CEREBROVASCULAR DISEASES	553	2.677	Q3
4	NEUROLOGICAL SCIENCES	21 (3.45 %)	3.83	Q2	LANCET	527	202.731	Q1
5	EUROPEAN JOURNAL OF NEUROLOGY	20 (3.29 %)	6.288	Q1	INTERNATIONAL JOURNAL OF STROKE	473	6.948	Q1
6	CEREBROVASCULAR DISEASES	16 (2.63 %)	3.104	Q3	JAMA NEUROLOGY	329	29.907	Q1
7	AMERICAN JOURNAL OF NEURORADIOLOGY	13 (2.14 %)	4.966	Q2	NEUROLOGY	290	12.258	Q1
8	INTERNATIONAL JOURNAL OF STROKE	11 (1.81 %)	6.948	Q1	JAMA-JOURNAL OF THE AMERICAN MEDICAL ASSOCIATION	276	157.375	Q1
9	JOURNAL OF NEUROINTERVENTIONAL SURGERY	10 (1.64 %)	8.572	Q1	JOURNAL OF THROMBOSIS AND HAEMOSTASIS	259	16.041	Q1
10	JOURNAL OF NEUROLOGY	10 (1.64 %)	6.682	Q1	EUROPEAN JOURNAL OF NEUROLOGY	253	6.288	Q1

NP, N of publications; IF, impact factor; JCR, Journal Citation Reports.

Furthermore, 10,342 references were cited in these 608 papers. These references were taken from 3,014 journals, with the top 10 publications cited over 250 times each. The most-cited journal was STROKE, followed by the NEW ENGLAND JOURNAL OF MEDICINE, JOURNAL OF STROKE and CEREBROVASCULAR DISEASES, LANCET, INTERNATIONAL JOURNAL OF STROKE, JAMA NEUROLOGY, NEUROLOGY, JAMA-JOURNAL OF THE AMERICAN MEDICAL ASSOCIATION, JOURNAL OF THROMBOSIS AND HAEMOSTASIS, and EUROPEAN JOURNAL OF NEUROLOGY (Table 1). Nine of these journals are ranked JCR Q1. Interestingly, the JOURNAL OF STROKE and CEREBROVASCULAR DISEASES (IF = 2.677, JCR Q3), STROKE (IF = 10.17, JCR Q1), EUROPEAN JOURNAL OF NEUROLOGY (IF = 6.288, JCR Q1), and INTERNATIONAL JOURNAL OF STROKE (IF = 6.948, JCR Q1) were simultaneously located in the top 10 most prolific journals and the top 10 most-cited journals, indicating that these four journals have made important contributions to COVID-19 and stroke research.

### 3.3. Top contributing countries/regions

**Figure 2:**
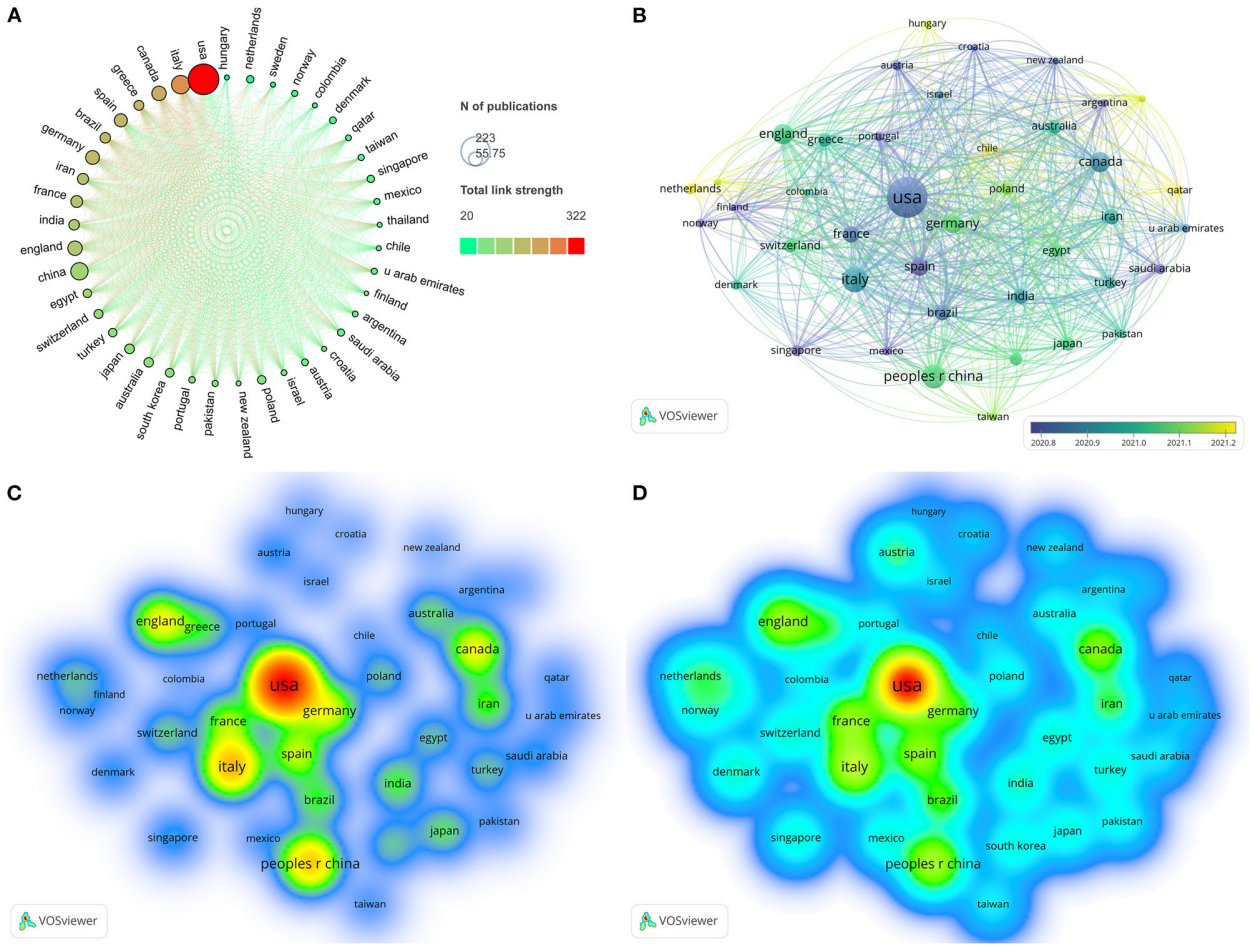
Country/region co-authorship networks. **(A)** Scimago Graphica-generated chord diagram of country/region cooperation network ranked by total link strength. The size of the nodes represents the number of publications of each country/region, and the color of the nodes represents the intensity of cooperation between countries/regions. **(B)** Dynamics and trends of the countries/regions with five publications or more. The size of the nodes represents the number of publications, and the color depth of the nodes represents the average appearance year of the publication. **(C)** Density map of the number of publications of countries/regions generated by VOSviewer. Both the color and font size indicates the number of publications. **(D)** Density map of the frequency of citation of countries/regions generated by VOSviewer. Both the color and font size indicates the citation frequency.

Researchers from 81 countries/regions contributed to COVID-19 and stroke studies. Table 2 displays the list of the ten most prolific countries/regions. The United States produced the most publications (223), followed by Italy (80), China (71), Canada (49), and England (49). We visualized the co-occurrence network of 41 countries/regions with at least five publications using the VOSviewer (Figure 2). In addition, the country/region co-occurrence network is imported into Scimago Graphica to generate a chord diagram. As shown in Figure 2A, the United States had the highest total link strength (TLS), followed by Italy, Canada, and Greece, indicating a high cooperation intensity in these countries/regions. In addition, Figure 2B shows the average time for publishing relevant studies from various countries/regions. Furthermore, Figures 2C, D displayed the density plots of the publication and citation counts for these nations/regions, respectively. We found that the United States had the highest collaboration intensity, the earliest initiation of research, the highest volume of publications, and the maximum number of citations. Consequently, American researchers have made significant contributions to COVID-19 and stroke-related studies.

**Table 2:** Top 10 most prolific countries in COVID-19 and stroke research.

**Rank**	**Countries/regions**	**NP**	**Citations**
1	USA	223	5,042
2	Italy	80	1,448
3	China	71	1,228
4	Canada	49	985
5	England	49	1,127
6	Germany	45	751
7	Spain	40	1,045
8	France	33	1,063
9	Iran	29	584
10	Brazil	26	690

NP, N of publications.

### 3.4. Top contributing authors

**Figure 3:**
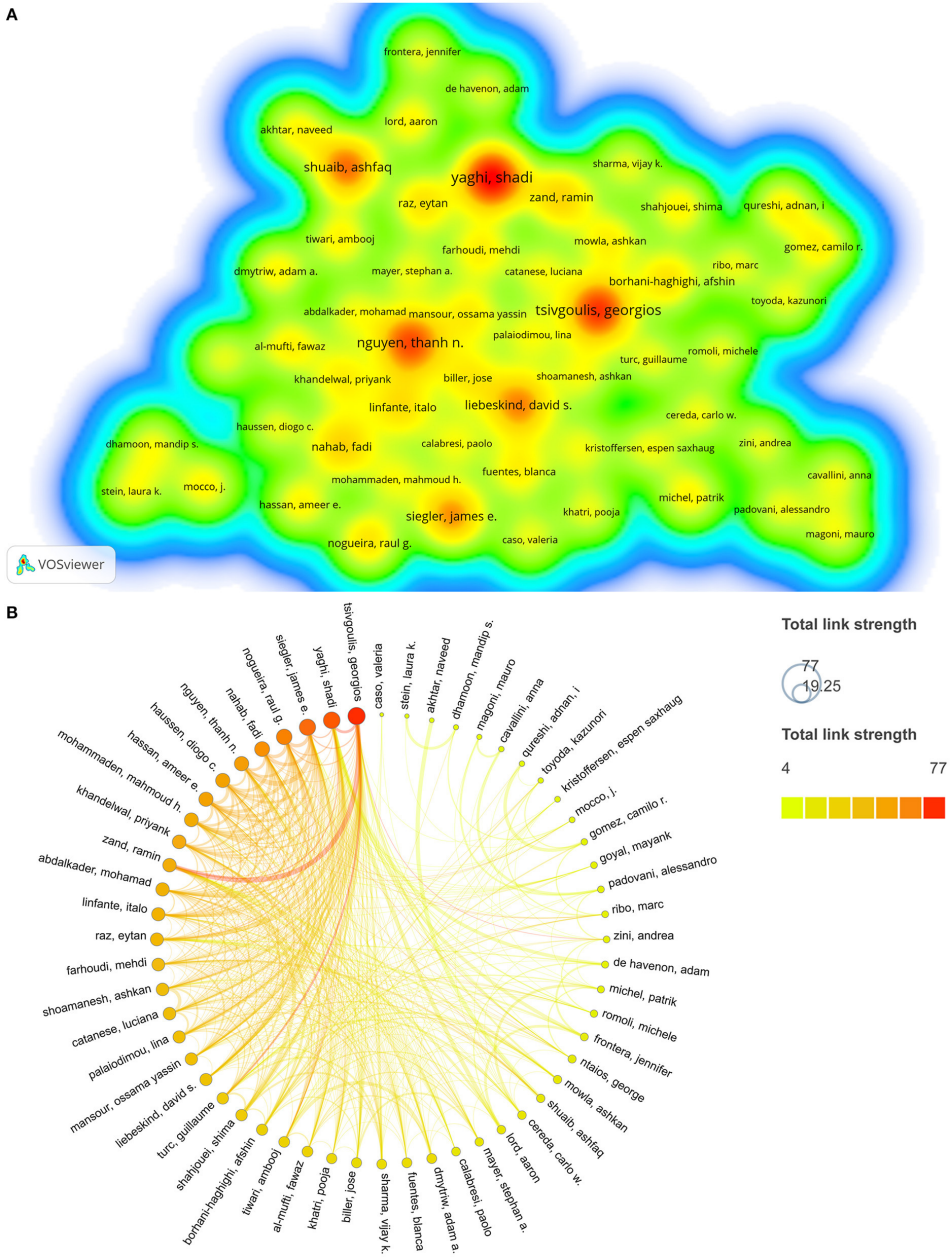
Author co-authorship networks. **(A)** Density map of the number of publications by author generated by VOSviewer. Both the color and font size indicates the number of publications of each author. **(B)** Scimago Graphica-generated chord diagram of author cooperation network. Both the color and size of the nodes indicate the intensity of cooperation between authors.

A total of 5,323 authors have contributed to the COVID-19 and stroke research field between 2020 and 2022. Table 3 displays the 12 most prolific authors in the field who have contributed at least seven publications. Shadi Yaghi's 15 publications have made substantial contributions to the field, followed by Georgios Tsivgoulis (13 publications), Thanh N Nguyen (12 publications), Ashfaq Shuaib (11 publications), David S Liebeskind (10 publications), and James E Siegler (10 publications). More than fifty percent of these prolific authors were American. The co-occurrence network of 56 authors with five or more publications was visualized using density plots in VOSviewer software (Figure 3A). The redder the color, the greater the number of publications; the closer the distance, the greater the collaboration intensity (Figure 3A). In addition, the network was imported into Scimago Graphica software to construct a chord diagram, with the node size set to indicate the total link strength (Figure 3B). Thicker connecting lines indicated greater author collaboration. In the co-occurrence network, Georgios Tsivgoulis had the strongest total link strength (total link strength = 77), followed by Shadi Yaghi (total link strength = 71), James E Siegler (total link strength = 68), Raul G Nogueira (total link strength = 62), and Fadi Nahab (total link strength = 57), indicating that these authors conducted more collaborations in the field of COVID-19 and stroke (Figure 3B). Intriguingly, both Georgios Tsivgoulis and Shadi Yaghi simultaneously ranked in the top five for the number of publications and overall link strength, showing their outstanding contributions to the subject.

**Table 3:** The most prolific author in the field of COVID-19 and stroke research.

**Rank**	**Author**	**NP**	**TC**	**CPP**	**Institution**	**Country**
1	Shadi Yaghi	15	781	52.07	New York University	USA
2	Georgios Tsivgoulis	13	289	22.23	Athens Medical School	Greece
3	Thanh N Nguyen	12	141	11.75	Boston University	USA
4	Ashfaq Shuaib	11	53	4.82	University of Alberta	Canada
5	David S Liebeskind	10	197	19.7	University of California Los Angeles	USA
6	James E Siegler	10	209	20.9	Cooper University Hospital	USA
7	Fadi Nahab	8	159	19.88	Emory University	USA
8	Ramin Zand	8	186	23.25	Geisinger Health System	USA
9	Afshin Borhani-Haghighi	7	284	40.57	Shiraz University of Medical Science	Iran
10	Italo Linfante	7	177	25.29	Linfante, Italo	USA
11	Raul G Nogueira	7	178	25.43	Emory University	USA
12	Eytan Raz	7	648	92.57	NYU Langone Medical Center	USA

NP, N of publications; TC, total citation; CPP, citation per publication.

### 3.5. Top contributing institutions

**Figure 4:**
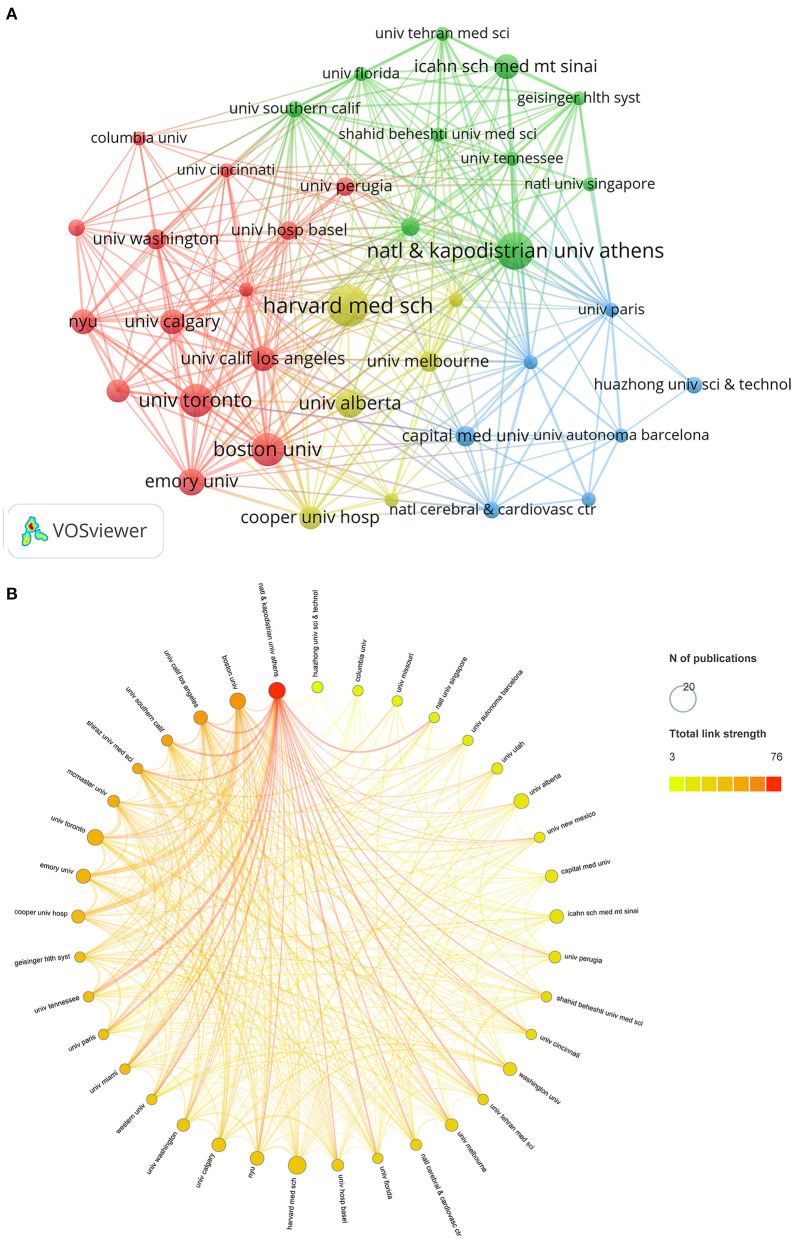
Institution co-authorship networks. **(A)** Co-occurrence network of institutions generated by VOSviewer. The size of the nodes represents the number of publications. The size of the nodes represents the number of publications of each institution, and the distance between nodes and the thickness of the link represents the intensity of cooperation between institutions. **(B)** Scimago Graphica-generated chord diagram of institution cooperation network. The size of the nodes represents the number of publications, and the color of the nodes represents the intensity of cooperation between institutions.

There are 1,911 institutions that have contributed to COVID-19 and stroke research. As shown in Table 4, the top 10 most prolific institutions are located in the United States (*n* = 6), Canada (*n* = 3), and Greece (*n* = 1). Harvard Medical School has the most publications in this field (*n* = 20), followed by the National and Kapodistrian University of Athens (*n* = 18), Boston University (*n* = 16), and the University of Toronto (*n* = 16). We constructed a cooperative co-occurrence network of institutions using the VOSviewer software and imported the network into the Scimago Graphica software to plot chord diagrams (Figure 4). Figure 4A displayed a collaborative network of 37 institutions with seven or more publications organized into four clusters. The size of the nodes represents the number of publications. The distance between nodes and the thickness of the link represents the strength of cooperation between institutions. National and Kapodistrian University of Athens has the strongest total link strength (total link strength = 76), indicating that they have the strongest collaboration with other institutions, followed by Boston University (total link strength = 55), University of California, Los Angeles (total link strength = 54), and University of Southern California (total link strength = 50) (Figure 4B).

**Table 4:** The most prolific institutions in the field of COVID-19 and stroke research.

**Institution**	**Location**	**NP**	**TC**	**TLS**
Harvard Medical School	USA	20	422	35
National and Kapodistrian University of Athens	Greece	18	429	76
Boston University	USA	16	188	55
University of Toronto	Canada	16	355	44
University of Alberta	Canada	14	67	19
Emory University	USA	13	231	43
Icahn School of Medicine at Mount Sinai	USA	12	371	22
New York University	USA	12	272	35
University of Calgary	Canada	12	319	35
University of California, Los Angeles	USA	12	249	54

NP, N of publications; TC, total citation; TLS, total link strength.

### 3.6. Top influential publications

Table 5 is a list of the top 20 most-cited publications. The most-cited publication is “SARS-CoV-2 and Stroke in a New York Healthcare System” published by Shadi Yaghi in STROKE. The second most frequently cited publication is “COVID-19 presenting as stroke” published by Akshay Avula in BRAIN BEHAVIOR AND IMMUNITY. The third most-cited publication is “Risk of Ischemic Stroke in Patients With Coronavirus Disease 2019 (COVID-19) vs. Patients With Influenza” published by Alexander E Merkler in JAMA NEUROLOGY. Interestingly, half of the top twenty most-cited works are published in STROKE. Nevertheless, it is crucial to remember that depending merely on the number of citations to determine the significance of a publication might be restrictive, as earlier-published works are more likely to have higher citation counts.

**Table 5:** Top 20 most-cited publications on COVID-19 and stroke.

**Rank**	**Title**	**First author**	**Publication year**	**Journal**	**IF (2021)**	**TC**
1	SARS-CoV-2 and Stroke in a New York Healthcare System	Shadi Yaghi	2020	STROKE	10.17	409
2	COVID-19 presenting as stroke	Akshay Avula	2020	BRAIN BEHAVIOR AND IMMUNITY	19.227	345
3	Risk of ischemic stroke in patients with coronavirus disease 2019 (COVID-19) vs. patients with influenza	Alexander E Merkler	2020	JAMA NEUROLOGY	29.907	299
4	COVID-19 and stroke: a global World Stroke Organization perspective	Hugh S Markus	2020	INTERNATIONAL JOURNAL OF STROKE	6.948	241
5	COVID-19-related stroke	David C Hess	2020	TRANSLATIONAL STROKE RESEARCH	6.8	228
6	Stroke in COVID-19: a systematic review and meta-analysis	Stefania Nannoni	2020	INTERNATIONAL JOURNAL OF STROKE	6.948	193
7	Impact of the COVID-19 epidemic on stroke care and potential solutions	Jing Zhao	2020	STROKE	10.17	191
8	Status of SARS-CoV-2 in cerebrospinal fluid of patients with COVID-19 and stroke	Fadi Al Saiegh	2020	JOURNAL OF NEUROLOGY NEUROSURGERY AND PSYCHIATRY	13.654	168
9	Acute stroke care is at risk in the era of COVID-19 experience at a comprehensive stroke center in Barcelona	Salvatore Rudilosso	2020	STROKE	10.17	164
10	Stroke in patients with SARS-CoV-2 infection: case series	Mauro Morassi	2020	JOURNAL OF NEUROLOGY	6.682	147
11	COVID-19 and ischemic stroke: a systematic review and meta-summary of the literature	Ying-Kiat Tan	2020	JOURNAL OF THROMBOSIS AND THROMBOLYSIS	5.221	141
12	Characteristics and Outcomes in Patients With COVID-19 and Acute Ischemic Stroke The Global COVID-19 Stroke Registry	George Ntaios	2020	STROKE	10.17	137
13	Decrease in hospital admissions for transient ischemic attack, mild, and moderate stroke during the COVID-19 era	Henrique Diegoli	2020	STROKE	10.17	133
14	Delays in stroke onset to hospital arrival time during COVID-19	Kay-Cheong Teo	2020	STROKE	10.17	132
15	Management of acute ischemic stroke in patients with COVID-19 infection: report of an international panel	Adnan I Qureshi	2020	INTERNATIONAL JOURNAL OF STROKE	6.948	132
16	Protected code stroke hyperacute stroke management during the coronavirus disease 2019 (COVID-19) pandemic	Houman Khosravani	2020	STROKE	10.17	131
17	Acute stroke in times of the COVID-19 pandemic a multicenter study	Carolin Hoyer	2020	STROKE	10.17	122
18	Coronavirus disease 2019 and stroke: clinical manifestations and pathophysiological insights	Afshin A Divani	2020	JOURNAL OF STROKE and CEREBROVASCULAR DISEASES	2.677	110
19	Treatment of acute ischemic stroke due to large vessel occlusion with COVID-19 experience from Paris	Simon Escalard	2020	STROKE	10.17	110
20	Mechanical thrombectomy for acute ischemic stroke amid the COVID-19 outbreak: decreased activity, and increased care delays	Basile Kerleroux	2020	STROKE	10.17	108

IF, impact factor; TC, total citation.

### 3.7. Analysis of co-occurring keywords

**Figure 5:**
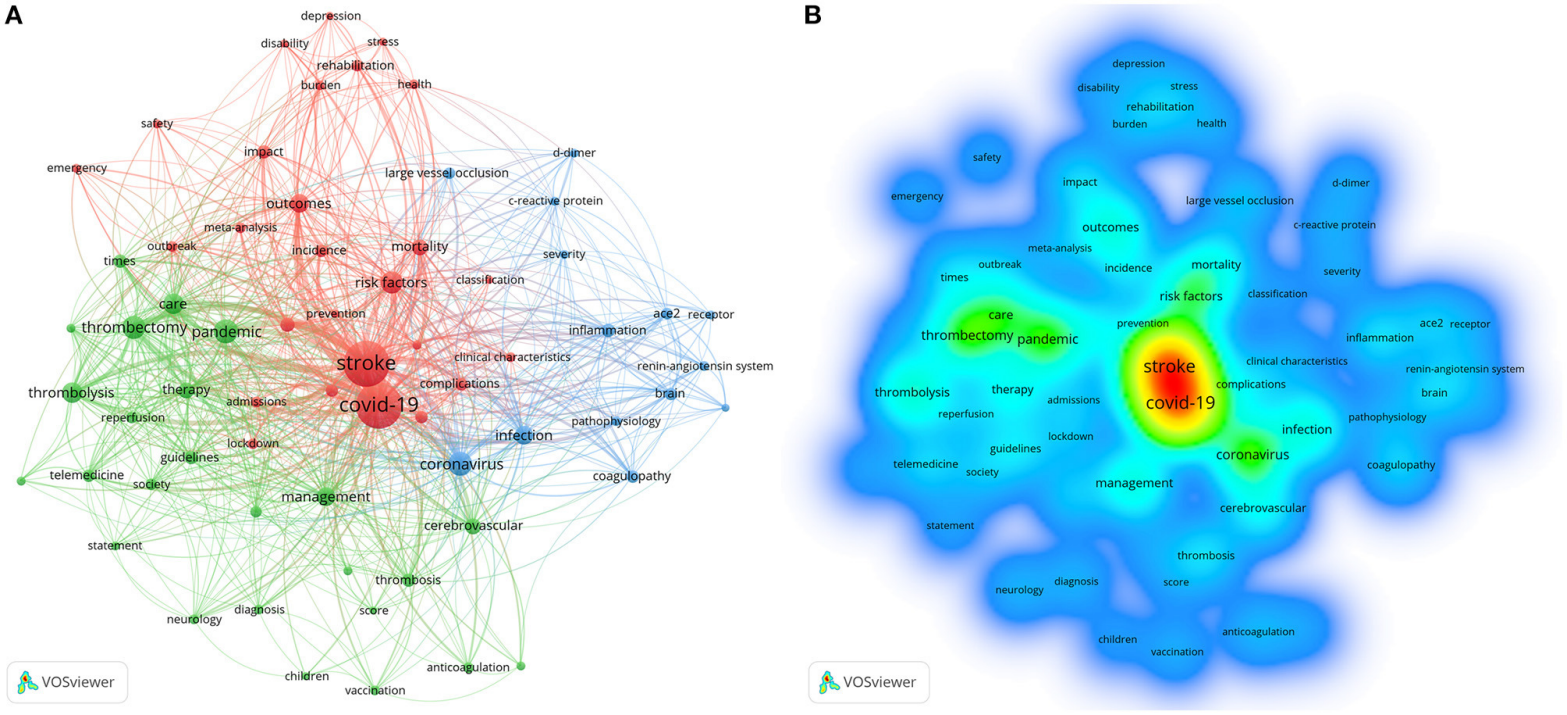
Sixty-six keywords with five or more occurrences were identified by VOSviewer. **(A)** Keyword co-occurrence network in COVID-19 and stroke studies. The size of the nodes represents the number of keyword occurrences, and the distance between the nodes and the thickness of the line between the nodes represent the frequency of concurrent occurrences of two keywords. **(B)** Density diagram of keywords in COVID-19 and stroke studies. Both the color and font size indicates the frequency of keyword occurrence.

The VOSviewer software was utilized to construct a co-occurrence network for 66 keywords that occurred at least five times (Figure 5). The network contains a total of 66 nodes and 776 links (Figure 5A). Table 6 displays the top twenty most prevalent keywords. Stroke was the most frequently occurring keyword (*n* = 469), followed by COVID-19 (*n* = 452), coronavirus (*n* = 87), pandemic (*n* = 81), thrombectomy (*n* = 78), risk factors (*n* = 66), care (*n* = 57), and thrombolysis (*n* = 53). In addition, these keywords are divided into 3 clusters: (i) cluster red: the impact of COVID-19 on stroke outcomes; (ii) cluster green: the management and care of stroke patients during the COVID-19 pandemic; and (iii) cluster blue: the potential relationship and pathological mechanism between COVID-19 and stroke. Furthermore, the density map revealed with greater prominence the frequency with which these keywords occurred (Figure 5B).

**Table 6:** Top 20 most frequent keywords.

**Rank**	**Keyword**	**Occurrences**	**Cluster**
1	Stroke	469	1
2	COVID-19	452	1
3	Coronavirus	87	3
4	Pandemic	81	2
5	Thrombectomy	78	2
6	Risk factors	66	1
7	Care	57	2
8	Thrombolysis	53	2
9	Infection	44	3
10	Management	43	2
11	Outcomes	43	1
12	Therapy	30	2
13	Mortality	29	1
14	Cerebrovascular	28	2
15	Epidemiology	23	1
16	Guidelines	20	2
17	Inflammation	19	3
18	Thrombosis	19	2
19	Times	19	2
20	Impact	18	1

### 3.8. Analysis of reference co-citation network

**Figure 6:**
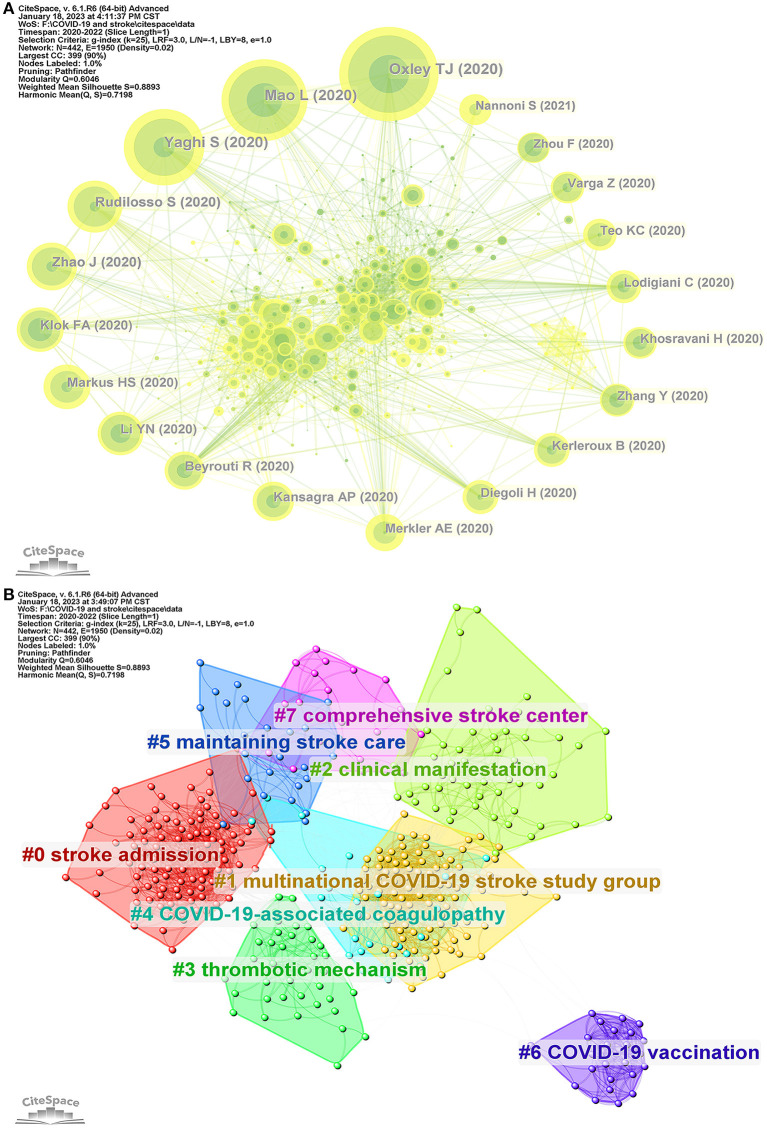
Analysis of cited references **(A)** Reference co-citation network. The size of the nodes represents citation frequency, and the thickness of the line represents co-citation frequency. **(B)** Eight reference clusters were identified by Citespace.

In 608 COVID-19 and stroke-related studies, 10,342 references were cited in total. These references provide a foundation for research in this field. We used the Citespace software to generate a co-citation network, which consisted of 442 nodes and 1950 links (Figure 6A). The size of the nodes denoted citation frequency, while the thickness of the line represented co-citation frequency. Table 7 lists the top 10 most-cited references. The most-cited reference by Thomas J. Oxley (2020), entitled “Large-Vessel Stroke as a Presenting Feature of COVID-19 in the Young,” was published in the NEW ENGLAND JOURNAL OF MEDICINE. In addition, the top 10 references by centrality are listed in Table 8. The cited reference ranking first by centrality was “Coronavirus disease 2019 and stroke in Iran: a case series and effects on stroke admissions,” which was published by M Mehrpour (2021) in the INTERNATIONAL JOURNAL OF STROKE.

**Table 7:** Top 10 most-cited references in COVID-19 and stroke-related studies ranked by total citations.

**Rank**	**Title**	**First author**	**Publication year**	**Journal**	**IF (2021)**	**TC**
1	Large-vessel stroke as a presenting feature of COVID-19 in the young	Thomas J. Oxley	2020	NEW ENGLAND JOURNAL OF MEDICINE	176.082	180
2	Neurologic manifestations of hospitalized patients with coronavirus disease 2019 in Wuhan, China	Ling Mao	2020	JAMA NEUROLOGY	29.907	156
3	SARS-CoV-2 and stroke in a New York healthcare system	Shadi Yaghi	2020	STROKE	10.17	145
4	Acute stroke care is at risk in the era of COVID-19 experience at a comprehensive stroke center in Barcelona	Salvatore Rudilosso	2020	STROKE	10.17	100
5	Impact of the COVID-19 epidemic on stroke care and potential solutions	Jing Zhao	2020	STROKE	10.17	91
6	COVID-19 and stroke: a global World Stroke Organization perspective	Hugh S Markus	2020	INTERNATIONAL JOURNAL OF STROKE	6.948	84
7	Incidence of thrombotic complications in critically ill ICU patients with COVID-19	F A Klok	2020	THROMBOSIS RESEARCH	10.409	84
8	Acute cerebrovascular disease following COVID-19: a single center, retrospective, observational study	Yanan Li	2020	STROKE AND VASCULAR NEUROLOGY	9.893	83
9	Collateral effect of COVID-19 on stroke evaluation in the United States	Akash P Kansagra	2020	NEW ENGLAND JOURNAL OF MEDICINE	176.082	74
10	Characteristics of ischaemic stroke associated with COVID-19	Rahma Beyrouti	2020	JOURNAL OF NEUROLOGY NEUROSURGERY AND PSYCHIATRY	13.654	74

IF, impact factor; TC, total citation.

**Table 8:** Top 10 cited references in COVID-19 and stroke-related studies ranked by centrality.

**Rank**	**Title**	**First author**	**Publication year**	**Journal**	**IF (2021)**	**Centrality**
1	Coronavirus disease 2019 and stroke in Iran: a case series and effects on stroke admissions	M Mehrpour	2021	INTERNATIONAL JOURNAL OF STROKE	6.948	0.13
2	Nervous system involvement after infection with COVID-19 and other coronaviruses	Yeshun Wu	2020	BRAIN BEHAVIOR AND IMMUNITY	19.227	0.1
3	Stroke in patients with SARS-CoV-2 infection: case series	Mauro Morassi	2020	JOURNAL OF NEUROLOGY	6.682	0.09
4	SARS-CoV-2 and stroke in a New York healthcare system	Shadi Yaghi	2020	STROKE	10.17	0.08
5	Acute cerebrovascular events in hospitalized COVID-19 patients	Aaron Rothstein	2020	STROKE	10.17	0.08
6	Acute stroke care is at risk in the era of COVID-19: experience at a comprehensive stroke center in Barcelona	Salvatore Rudilosso	2020	STROKE	10.17	0.07
7	Treatment of acute ischemic stroke due to large vessel occlusion with COVID-19: experience from Paris	Simon Escalard	2020	STROKE	10.17	0.07
8	Stroke code presentations, interventions, and outcomes before and during the COVID-19 pandemic	Adam S Jasne	2020	STROKE	10.17	0.07
9	Pulmonary vascular endothelialitis, thrombosis, and angiogenesis in COVID-19	Maximilian Ackermann	2020	NEW ENGLAND JOURNAL OF MEDICINE	176.082	0.07
10	Endovascular thrombectomy in acute ischemic stroke patients with COVID-19: prevalence, demographics, and outcomes	Adam de Havenon	2020	JOURNAL OF NEUROINTERVENTIONAL SURGERY	8.572	0.07

IF, impact factor.

Furthermore, we performed a clustering analysis of the reference co-citation network using Citespace. Figure 6B shows eight clusters of the co-citation network. Each cluster has many cited references that are closely related. The clusters are designated as follows: #0 stroke admission, #1 multinational COVID-19 stroke study group, #2 clinical manifestation, #3 thrombotic mechanism, #4 COVID-19-associated coagulopathy, #5 maintaining stroke care, #6 COVID-19 vaccination, and #7 comprehensive stroke center.

## 4. Discussion

Overall, we identified the most influential journals, countries/regions, authors, institutions, and publications by conducting a bibliometric analysis of 608 publications regarding COVID-19 and stroke from January 2020 to December 2022. In addition, we identified the most active research topics in this field by performing keyword co-occurrence analysis and reference co-citation analysis.

With 12.50% of relevant articles published, the JOURNAL OF STROKE and CEREBROVASCULAR DISEASES is one of the most prominent journals in COVID-19 and stroke research. In addition to being the most prolific journal in this field, it is also the third most-cited journal among relevant papers. Another prominent journal on COVID-19 and stroke is STROKE, which has the second-largest number of pertinent publications and the highest citation rate. In addition, the EUROPEAN JOURNAL OF NEUROLOGY and the INTERNATIONAL JOURNAL OF STROKE were simultaneously in the top 10 most prolific journals and top 10 most frequently cited journals.

According to the findings of this bibliometric analysis, the United States is the leading contributor to COVID-19 and stroke research, with a substantial number of publications and citations. This is likely attributable to the country's robust research infrastructure and funding opportunities. Furthermore, the high collaboration intensity between institutions and researchers in the USA suggests a strong and active research community in this field.

In terms of top contributing authors, our analysis identified several key individuals who have made substantial contributions to the field, with many publications and collaborations with their peers. The fact that Georgios Tsivgoulis and Shadi Yaghi concurrently ranked in the top five for the number of publications and total link strength indicates their remarkable contributions to the field.

The top contributing institutions were predominantly American, with Harvard Medical School producing the most articles in this field. The collaborative network of institutions also revealed several clusters, indicating different research areas and collaborations within the field. These results suggest that these institutions have played a significant role in advancing the knowledge and understanding of COVID-19 and stroke.

We identify the most influential publications based on the number of citations. The article “SARS-CoV-2 and Stroke in a New York Healthcare System” by Shadi Yaghi, published in the journal STROKE, is the most-cited publication in the field of COVID-19 and stroke, with 409 citations. The study indicated that stroke patients with COVID-19 had much poorer outcomes than those without the infection, including higher scores on the National Institutes of Health Stroke Scale, higher peak D-dimer levels, and significantly greater mortality.

In prior research, Zhang et al. (15) employed bibliometric analysis to investigate publications on COVID-19 in the field of neurology, while Chan et al. (14) conducted a similar study using bibliometrics to explore the 100 most frequently cited articles on COVID-19 in the fields of neurology and neurosurgery. Interestingly, some of our findings are similar to these two studies. USA and Italy were identified as the top two countries with the highest number of publications across all three studies for several possible reasons: (i) The high prevalence of COVID-19 in the United States and Italy has led to greater interest and urgency in understanding the effects of COVID-19 on the brain and nervous system. (ii) As developed countries, both countries have well-established healthcare systems and research institutions with a strong focus on neurology and stroke research, and these institutions have the necessary resources, expertise, and funding to conduct research and publish in these areas. (iii) The COVID-19 pandemic has led to a global collaboration to understand and combat the virus. The United States and Italy have been active participants in this collaboration, leading to an increase in the number of joint publications with researchers from other countries. In addition, consistent with the two previous publications, Harvard Medical School in the USA was the most prolific institution, which may be attributed to the combination of a rich history of excellence in medical research and education, a diverse and talented faculty, strong funding and infrastructure, and a collaborative research culture. In terms of research trends, both our study and Zhang et al.'s study identified potential mechanisms of COVID-19-induced neurological disorders and cerebrovascular diseases, highlighting the impact of COVID-19-induced inflammatory storms on the brain. Nevertheless, certain research trends identified in this study differed from previous studies. Since we were more focused on the impact of COVID-19 on stroke, trends such as thrombectomy and thrombolysis management during the COVID-19 epidemic were highlighted as one of the research hotspots.

The keyword co-occurrence network created by VOSviewer divides keywords into three main groups, providing greater insight into specific areas of focus within the field. The keywords in Cluster red, “the impact of COVID-19 on stroke outcomes,” reveal a focus on the effects of COVID-19 on stroke outcomes. This cluster also includes keywords related to complications, incidence, and risk factors, indicating that researchers have also been interested in identifying specific factors that may contribute to worse outcomes in stroke patients with COVID-19. Cluster green, which includes keywords related to “management and care of stroke patients during the COVID-19 pandemic,” such as “thrombectomy,” “care,” “thrombolysis,” “guidelines,” and “telemedicine,” suggests that a significant portion of the research has been dedicated to understanding how to best manage and treat stroke patients during the pandemic. Keywords such as “vaccination” and “public health” indicate that researchers have also been interested in understanding the role of vaccinations and public health in managing stroke patients during COVID-19. Cluster blue, which includes keywords related to “the potential relationship and pathological mechanisms between COVID-19 and stroke,” such as “coronavirus,” “infection,” “inflammation,” “brain,” “coagulopathy,” and “ACE2,” suggests that a significant portion of the research has been dedicated to understanding the underlying mechanisms that may link the two conditions. Keywords such as “pathophysiology,” “c-reactive protein,” and “d-dimer” indicate that researchers have also been interested in understanding the biomarkers and potential diagnostic tools that may be used to identify patients at risk for stroke during COVID-19. Overall, the analysis of co-occurring keywords provides insight into the main themes and topics studied in the COVID-19 and stroke and highlights the areas where further research is needed.

The top 10 most-cited references and the top 10 references ranked by centrality identified by Citespace can be classified into three main categories. The first category is related to neurological manifestations and the incidence of stroke in COVID-19 patients. The second category is about the impact of COVID-19 on stroke care. The third category is centered on thrombotic complications and treatment in COVID-19 patients. In addition, the reference co-citation network was classified into eight clusters: #0 stroke admission, #1 multinational COVID-19 stroke study group, #2 clinical manifestation, #3 thrombotic mechanism, #4 COVID-19-associated coagulopathy, #5 maintaining stroke care, #6 COVID-19 vaccination, and #7 comprehensive stroke center. Combining the results of keyword co-occurrences and reference co-citations, we found several research highlights of COVID-19 and stroke. The first research hotspot is the impact of the COVID-19 pandemic environment on the hospitalization of stroke patients, including more severe clinical symptoms and poorer prognoses. The second research hotspot focuses on the standardized care and treatment of stroke patients during the COVID-19 pandemic. The third research hotspot is the potential pathological mechanism of exacerbation of stroke attributed to COVID-19.

The influence of the COVID-19 pandemic on stroke outcome is, without a doubt, the first significant focus of early study. Numerous studies have discovered a link between COVID-19 and an elevated risk of stroke. For example, the article entitled “Risk of Ischemic Stroke in Patients With Coronavirus Disease 2019 (COVID-19) vs. Patients With Influenza” published in JAMA NEUROLOGY, indicated in the early stages that COVID-19 patients have a higher risk of stroke than influenza patients (6). In addition, the COVID-19 epidemic is associated with worse outcomes for stroke patients. For example, “Impact of the COVID-19 Epidemic on Stroke Care and Potential Solutions” and “Mechanical Thrombectomy for Acute Ischemic Stroke Amid the COVID-19 Outbreak: Decreased Activity, and Increased Care Delays” found a reduction in hospital admissions for stroke patients and a reduction in the volume of thrombolysis procedures during the COVID-19 epidemic (18, 19). These studies demonstrated the necessity of examining COVID-19 and stroke studies and finding corresponding solutions to optimize the prognosis of stroke patients during the epidemic.

The second major field is the standardized care and treatment of stroke inpatients during the COVID-19 epidemic. A large retrospective multicenter cohort study entitled “Safety and Outcome of Revascularization Treatment in Patients With Acute Ischemic Stroke and COVID-19: The Global COVID-19 Stroke Registry” reported that acute ischemic stroke patients with COVID-19 exhibited higher rates of intracranial hemorrhage complications and poorer clinical outcomes after revascularization therapy compared with non-COVID-19 infected acute ischemic stroke patients (20). However, current evidence does not provide enough information to conclude the effectiveness of revascularization treatments for COVID-19 patients with ischemic stroke or to provide specific treatment recommendations for this subgroup. Thus further research is needed to determine the best treatment for these patients. In addition, the importance of telemedicine in stroke became apparent due to the global occupation of medical resources during the epidemic. The article “Telestroke: Maintaining Quality Acute Stroke Care During the COVID-19 Pandemic” shows that telemedicine systems have provided uninterrupted stability of acute stroke care and treatment during a pandemic (21). The study demonstrates the effectiveness of telemedicine services in providing timely and high-quality stroke care, especially during a global health crisis. Furthermore, “Telerehabilitation of post-stroke patients as a treatment option in the era of the Covid-19 pandemic” emphasized the importance of telerehabilitation during the epidemic (22). Therefore, further research on the impact of telemedicine on neuro-intervention procedures and a comparison of outcomes between telemedicine and in-person consultations would be valuable.

The third major area of research is the potential pathological mechanism of exacerbation of stroke attributed to COVID-19. The underlying mechanism of stroke in COVID-19 patients is not well understood, but previous studies suggest it may be due to cerebrovascular pathology following viral infection, inflammation-induced endothelial dysfunction, and hypercoagulability (23). Inflammation-induced damage to the endothelium can trigger a coagulation cascade, resulting in thrombosis, destabilization of atherosclerosis plaques, and occlusion of large vessels, leading to ischemic stroke (24). In addition, the SARS-CoV-2 virus decreases the level of ACE2, which leads to increased AngII, which, in turn, activates the AngII type 1 receptor, leading to the production of inflammatory cytokines such as IL-6, TNF-α, MCP-1, and IL-8 through the NF-κB signaling pathway, causing acute endothelial dysfunction and immune-mediated injury (25). Furthermore, it has been observed that patients diagnosed with COVID-19 are at a greater risk of developing a hypercoagulable state within their vasculature (26). Research indicates that these individuals exhibit elevated levels of factor VIII, fibrinogen, and d-dimer, which are all directly involved in the clotting process (27). It should be emphasized that, despite keyword clustering and reference co-citation analysis identifying this area of research, the top 20 cited papers on this subject are limited. Therefore, this indicates that the field may be the focus of future investigations.

This study has some limitations that should be considered. First, the scope of the study is limited to articles published in the English language. Therefore it is possible that some relevant literature in other languages may not have been included. Second, the bibliometric analysis only provides a quantitative overview and does not reflect the quality or significance of the studies included. Third, in addition to WoSCC, bibliometric analysis can utilize databases like Scopus and PubMed. However, the data collection policies of these databases can impact the scope and citation count of publications. Despite their potential value, integrating multiple databases into a single bibliometric analysis can be difficult due to differences in citation indexes and metadata structures. This challenge has also been observed in other bibliometric research studies (28). Finally, since WoSCC would not index articles immediately after publication, a small number of new future indexed publications were not included in this study.

## 5. Conclusion

Overall, this bibliometric analysis provides a comprehensive overview of the current state of research on COVID-19 and stroke and highlights critical areas of focus for future research in this field. Optimizing the treatment of COVID-19-infected stroke patients and elucidating the underlying pathogenic mechanisms of COVID-19 and stroke are crucial research areas that must be pursued further to enhance outcomes for stroke patients during the continuing COVID-19 pandemic.

## Data availability statement

The original contributions presented in the study are included in the article/supplementary material, further inquiries can be directed to the corresponding author.

## Author contributions

YZ designed the study, analyzed the data, and wrote the manuscript. SC assisted in analyzing the data and revising the manuscript. HY critically read and edited the manuscript. All authors contributed to the article and approved the submitted version.
